# Direction of Association between Bite Wounds and *Mycobacterium bovis* Infection in Badgers: Implications for Transmission

**DOI:** 10.1371/journal.pone.0045584

**Published:** 2012-09-19

**Authors:** Helen E. Jenkins, D. R. Cox, Richard J. Delahay

**Affiliations:** 1 Medical Research Centre for Outbreak Analysis and Modelling, Department of Infectious Disease Epidemiology, Imperial College London, London, United Kingdom; 2 Department of Statistics, Nuffield College, University of Oxford, Oxford, United Kingdom; 3 Wildlife and Emerging Disease Programme, The Food and Environment Research Agency, York, United Kingdom; University of Pittsburgh, United States of America

## Abstract

**Background:**

Badgers are involved in the transmission to cattle of bovine tuberculosis (TB), a serious problem for the UK farming industry. Cross-sectional studies have shown an association between bite wounds and TB infection in badgers which may have implications for *M. bovis* transmission and control, although the sequence of these two events is unclear. Transmission during aggressive encounters could potentially reduce the effectiveness of policies which increase the average range of a badger and thus its opportunities for interaction with other social groups.

**Methods:**

Data were obtained on badgers captured during a long term study at Woodchester Park, UK (1998–2006). Many badgers had multiple observations. At each observation, the badger was assigned a “state” depending on presence of bite wounds and/or TB infection. Hence each badger had a “transition” from the previous state to the current state. We calculated the numbers of each type of transition and the time spent in each state. Transition rates were calculated for each transition category, dividing the number of such transitions by the total time at risk. We compared the rate of bite wound acquisition in infected badgers with that for uninfected badgers and the rate of positive *M.bovis* test results in bitten badgers with that in unbitten badgers.

**Results:**

The rate of bite wound acquisition in infected badgers (0.291 per year) was 2.09 (95% CI: 1.41, 3.08) times that in uninfected badgers (0.139 per year). The rate of positive *M.bovis* test results in bitten badgers (0.097 per year) was 2.45 (95% CI: 1.29, 4.65) times that in unbitten badgers (0.040 per year).

**Conclusions:**

We found strong evidence of both potential sequences of events consistent with transmission via bite wounds and distinctive behaviour in infected badgers. The complex relationship between behaviour and infection must be considered when planning TB control strategies.

## Introduction

Bovine tuberculosis (TB) is a serious problem for cattle farmers in the U.K. and Republic of Ireland [1], [2]. Badgers (*Meles meles*) also can be infected with *Mycobacterium bovis* (*M.bovis*), the causative agent for TB and are an important potential source of infection for cattle [3], [4]. While a recent large-scale field experiment in the UK provided evidence of transmission of *M.bovis* between both host species [4]–[6], the utility of badger culling in reducing TB in cattle is unclear [5], [7]. Furthermore, evidence from several field studies shows that culling can result in substantial disruption of badger social organization, which may provide enhanced opportunities for *M.bovis* transmission amongst badgers and between badgers and cattle [8]. Further understanding of the transmission dynamics of *M.bovis* in badgers and which groups of badgers may be at increased risk of infection may assist in the development of effective disease control methods.

Aerosol inhalation is considered the principal route of transmission of *M.bovis* between badgers [9]–[12]. However, there is also evidence of an association between bite wounds and *M.bovis* infection [11], [13], [14], implying an additional process that could be occurring alongside aerosol transmission. This additional hypothesis is that TB infected badgers may be more likely to be bitten. No studies to date have attempted to determine the likely relative importance of these two hypotheses. The direction of effect may have important implications for TB transmission. Previous work has shown that management interventions can have profound effects on the social behavior of badger populations [6], and hence potentially on rates of bite wounding. Consequently, further understanding of the relationship between biting and infection dynamics may contribute to the development of sustainable strategies to manage TB in badger populations. Here we use time series data from live wild badgers examined during a long-term capture-mark-recapture study, to answer two related questions:

1. Does the rate of being bitten differ between TB positive and negative badgers?
2. Does the rate of being detected as TB positive differ between badgers that have been bitten and those that have not?

## Methods

### Data Collection

The data analysed were from an intensively studied badger population at Woodchester Park, Gloucestershire, UK. The resident badgers were routinely trapped, marked with a unique tattoo, examined and released throughout the year (apart from February to April inclusive) from January 1998 to August 2006. Captured animals were examined for any bites and their age, sex and body condition recorded. Experienced staff were able to distinguish bites from other injuries by their characteristic size and shape and the presence of tooth puncture marks. TB infection status was determined in two ways, by culture of clinical samples (sputum, faeces, urine, abscess and wound swabs) [12] and by the Brock ELISA serological test [15]. The isolation by culture of *M.bovis* from clinical samples indicated that an individual was shedding bacteria and hence infectious. A positive response to the ELISA test indicates the presence of antibodies which potentially corresponds to active or latent infection. The culture of clinical samples has low sensitivity to detect infection (8% compared to 37% for Brock ELISA [15], [16]) but is highly specific [16] and persistent culture positivity is associated with reduced survival [17] (adjusting for age), suggesting that this is identifying animals at an advanced stage of disease progression. Hence, in this study we assumed that a culture positive result implied a more advanced stage of disease progression than an ELISA positive result [17], [18].

Animals were captured and examined under appropriate licenses issued by Natural England and the UK Home Office. Animals were examined in accordance with the Animals (Scientific Procedures) Act 1986 and under veterinary supervision. All work adhered to the highest possible standards of animal welfare and was cleared by the Food and Environment Research Agency Ethical Review Process prior to commencement.

### Statistical Analysis

The unit of analysis was a capture observation, and there were multiple observations for many of the badgers in the dataset. At each such observation, the badger was given a “status” label (Table 1) and a “transition” label was given as follows:

- Blank – if this was the first observation of a badger
- “Previous status” to “current status” for example if the badger was TB positive on a given occasion but at the previous observation was TB negative and had never had a bite wound recorded then the transition would be “N to TB-Cul”

**Table 1:** Status labels given to each badger at each of its observations.

Status	Status label	Culture positive	ELISA positive	Fresh bite wound	Earlier recorded bite wound and recovered
No earlier bite recorded or fresh bite woundor positive culture or ELISA result	N				
Earlier bite wound recorded	Bit P				✓
Fresh bite wound found	Bit C			✓	
Culture positive	TB-Cul	✓			
ELISA positive	TB-El		✓		
Culture positive and with a fresh bite wound	TB-Cul/Bit C	✓		✓	
ELISA positive and with a fresh bite wound	TB-El/Bit C		✓	✓	
Culture positive with an earlier bitewound recorded	TB-Cul/Bit P	✓			✓
ELISA positive with an earlier bitewound recorded	TB-El/Bit P		✓		✓

The check marks indicate the bite wound status and test results that define each status label.

At each observation, the time duration between the current and previous observation was calculated. It was assumed that the change in status occurred at the mid-point during this interval and hence half the duration time between observations was assigned to each state (current and previous). If there was no change in status between consecutive observations, the total duration time was assigned to that state. Across all badgers and all observations, the frequency of occurrence of each possible transition and the total time spent in each state were calculated.

Transition rates were calculated for each transition as the number of such transitions that occurred divided by the total time during which such a transition might have occurred. The number of transitions in each “transition” group was assumed to follow a Poisson distribution for which variances and confidence intervals for the log rate ratios were calculated. The implicit assumption was that the transition rates were not strongly age-dependent or having consistent changes in time. However, since the epidemiology of disease and the behaviour of badgers is markedly different in cubs and yearlings as compared with adults [6], [19], all observations that occurred in the first two years of life were excluded (2474 observations excluded). Our analyses were repeated for these excluded observations for comparison and are commented on in the discussion.

Once a badger returned an ELISA positive result, it was assumed in the analysis to remain ELISA positive unless it was subsequently found to be culture positive, in which case its infection status changed to culture positive and remained as such thereafter. Hence, in terms of infection status, badgers could go one of three routes: (1) from negative to ELISA positive and remain so until the end of the study period, (2) from negative to ELISA positive and then to culture positive, or (3) from negative directly to culture positive. The main focus of our two questions related to badgers that were known to be infected, therefore, we used culture positive badgers to answer these questions – those in routes (2) and (3). Owing to greater ambiguity over the predictive value of a positive ELISA test result to detect active disease, those animals in route (1) were classed as ELISA positive and treated separately and only those in routes (2) and (3) were included in the main analysis. Badgers in route (3) were considered infected at the mid-point between the first time that they had a culture positive result and the previous culture negative capture time. Since animals identified as route (2) later tested positive for TB by culture, we assumed that their earlier positive ELISA result was an early indication of infection. Therefore, for the purposes of this analysis, these badgers were retrospectively classed as TB infected at the time that they first became ELISA positive and all route (2) badgers were grouped with those in route (3). As with route (3), those badgers in route (2) were considered to be infected at the mid-point between the time of the first ELISA positive test and the time of the previous ELISA negative test.

Bite wounds found during the visual examination of anaesthetized captured badgers were defined as: “new”, “open (fresh)”, “old”, “healed (scar)” or “open (old)” (the latter indicating an old wound that never healed properly or had reopened) and the wound location on the body was also recorded. “Fresh” bite wound status was assigned to bites in the first two categories, and in the last three if it was the first bite wound recorded in that location on that animal. The latter definition of a fresh bite assumed that the bite had occurred since the previous capture but sufficient time had elapsed for the wound to heal. If the bite was in the last three categories but there was an indication at an earlier capture that the animal had a wound on that part of the body, the wound was assumed to be old and the badger assigned “earlier” bite wound status for that observation. If a badger was found to have a bite wound at one observation, it was classified as “earlier bite” at all subsequent captures, apart from when a fresh bite wound was observed (when it was classified as “fresh bite” for that observation).

Analyses were stratified by sex to check for the similarity of conclusions for males and females.

### Sensitivity Analyses

Since multiple observations could be included for an individual badger, we did two sensitivity analyses. This was to assess the robustness of our conclusions despite the presence of multiple observations per badger and to assess the impact of this lack of independence of observations on our standard errors. In the first analysis, we included only those badgers observed just twice so that only one transition would be included for each badger, thereby eliminating the issue of multiple observations. Since this first analysis could leave us with only a small total number of observations we did a second analysis. Here, we divided all of the observations into two approximately equal sized groups such that the first group contained badgers with 1, 2, etc. observations and the second group contained [the maximum number of observations], [the second highest number of observations], etc. We then looked for similar trends in both groups as were seen in the main results. To check that the multiple observations were not resulting in under-estimated standard errors, we compared the standard errors from the estimated transition rates in these two groups with those in the main analysis. If all observations on the same badger were independent of the rates, we would expect the standard errors in each of the two groups to be approximately √2 = 1.41 times those in the main analysis. If the standard errors had been found to be substantially higher than that then we would have concluded that internal correlation was under-estimating the standard errors and that some adjustment may be necessary.

We also stratified observations by age at capture to look for any age dependency in our results. Since stratifying by one year categories resulted in several categories with very few observations, we divided the data into two approximately equal sized groups as described above such that the first group contained observations from badgers aged 2 (years), 3 etc and the second group contained [the maximum age of the badgers], [the second highest age class of the badgers], etc. We then looked for similar trends in both groups as were seen in the main results.

Since specimens for culture were taken from bite wounds, where they were present, badgers with fresh bite wounds were more likely to have a positive culture result based on the fact that they had an additional site from which to obtain samples. To assess whether this had any impact on our results, we carried out a sensitivity analysis excluding all culture results obtained from bite wounds.

## Results

Data were available for 613 adult badgers (i.e. >2 years of age) captured between 1^st^ January 1998 and 31^st^ August 2006 (4263 observations in total), although only 400 were included in the analysis, as the remaining 213 animals had only one observation (capture) each. The maximum number of observations for a single badger was 28, and the median was 3 (excluding those badgers with only one observation each). Of those animals included in the analysis, 21.8% were detected as culture positive by clinical sampling (regardless of ELISA status), 27.5% at some point yielded a positive ELISA test result and 53.0% were observed with a bite wound at some point. A total of 18.3% of badgers were TB positive by culture at some point (regardless of ELISA status) and also had at least one bite wound, either before, afterwards or concurrently. All possible transition rates were calculated (Table 2).

**Table 2:** Annual rates of each possible transition.

Transition*	Previous bite status	Previous infection status	Current bite status	Current infection status	N	Time at risk (years)	Annual rate (Transitions (N) per year at risk)	Approx. 95% Confidence Interval
Bit C to Bit C	Fresh bite	None	Fresh bite	None	9	31.10	0.289	0.151, 0.556
Bit C to Bit P	Fresh bite	None	Earlier bite recorded	None	45	31.10	1.477	1.081, 1.938
Bit C to TB-Cul/Bit C	Fresh bite	None	Fresh bite	Culture +	2	31.10	0.064	0.016, 0.257
Bit C to TB-Cul/Bit P	Fresh bite	None	Earlier bite recorded	Culture +	2	31.10	0.064	0.016, 0.257
Bit C to TB-El/Bit P	Fresh bite	None	Earlier bite recorded	ELISA +	1	31.10	0.032	0.005, 0.228
N to Bit C	None	None	Fresh bite	None	12	352.13	0.034	0.019, 0.060
N to Bit P	None	None	Earlier bite recorded	None	47	352.13	0.133	0.100, 0.178
N to TB-Cul	None	None	None	Culture +	14	352.13	0.040	0.024, 0.067
N to TB-Cul/Bit P	None	None	Earlier bite recorded	Culture +	1	352.13	0.003	0.000, 0.020
N to TB-El	None	None	None	ELISA +	16	352.13	0.045	0.028, 0.074
N to TB-El/Bit C	None	None	Fresh bite	ELISA +	1	352.13	0.003	0.000, 0.020
N to TB-El/Bit P	None	None	Earlier bite recorded	ELISA +	1	352.13	0.003	0.000, 0.020
Bit P to Bit C	Earlier bite recorded	None	Fresh bite	None	21	256.44	0.082	0.053, 0.126
Bit P to TB-Cul/Bit C	Earlier bite recorded	None	Fresh bite	Culture +	4	256.44	0.016	0.006, 0.042
Bit P to TB-Cul/Bit P	Earlier bite recorded	None	Earlier bite recorded	Culture +	20	256.44	0.078	0.050, 0.121
Bit P to TB-El/Bit C	Earlier bite recorded	None	Fresh bite	ELISA +	1	256.44	0.004	0.001, 0.028
Bit P to TB-El/Bit P	Earlier bite recorded	None	Earlier bite recorded	ELISA +	12	256.44	0.047	0.027, 0.082
TB-Cul to TB-Cul/Bit C	None	Culture +	Fresh bite	Culture +	4	41.52	0.096	0.036, 0.257
TB-Cul to TB-Cul/Bit P	None	Culture +	Earlier bite recorded	Culture +	9	41.52	0.217	0.113, 0.417
TB-Cul/Bit C to TB-Cul/Bit C	Fresh bite	Culture +	Fresh bite	Culture +	5	11.08	0.451	0.188, 1.084
TB-Cul/Bit C to TB-Cul/Bit P	Fresh bite	Culture +	Earlier bite recorded	Culture +	20	11.08	1.805	1.165, 2.798
TB-Cul/Bit P to TB-Cul/Bit C	Earlier bite recorded	Culture +	Fresh bite	Culture +	17	67.81	0.251	0.156, 0.403
TB-El to TB-El/Bit C	None	ELISA +	Fresh bite	ELISA +	3	90.28	0.033	0.011, 0.103
TB-El to TB-El/Bit P	None	ELISA +	Earlier bite recorded	ELISA +	7	90.28	0.078	0.037, 0.163
TB-El/Bit C to TB-El/Bit P	Fresh bite	ELISA +	Earlier bite recorded	ELISA +	7	5.66	1.238	0.590, 2.597
TB-El/Bit P to TB-El/Bit C	Earlier bite recorded	ELISA +	Fresh bite	ELISA +	5	62.25	0.080	0.033, 0.193

* Note that some transitions occurred with very low frequency. They are included here for information although the estimated annual rates are very approximate.

**Figure 1:**
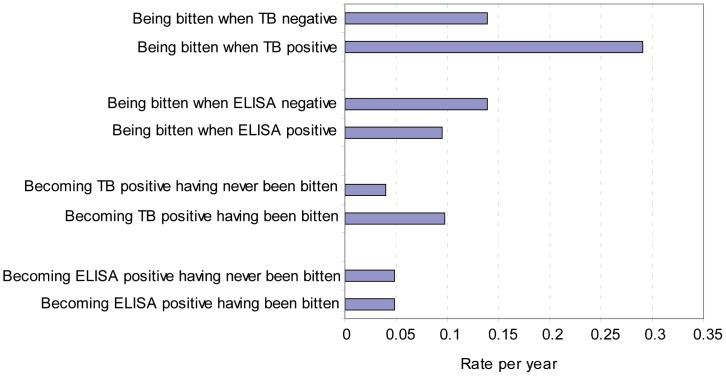
Annual rates of acquiring a bite wound, becoming TB (culture) positive and becoming ELISA positive, adults (badgers >2 years old) only.

In answer to question (1) (i.e. does the rate of being bitten differ between TB positive and negative badgers?), TB positive badgers (by culture) were significantly more likely to be bitten than TB negative badgers: rate ratio = 2.09 (95% CI: 1.41, 3.08, p<0.001) (Table 3, Figure 1). In answer to question (2) (i.e. does the rate of being detected as TB positive differ between badgers that have been bitten and those that have not?), badgers that had been bitten were more likely to be infected with *M.bovis* (as detected by culture) than badgers that had not been bitten: rate ratio = 2.45 (95% CI: 1.29, 4.65, p = 0.009) (Table 3, Figure 1).

**Table 3:** Annual rates of acquiring a bite wound, becoming culture positive and becoming ELISA positive.

Initial state of badger	Transition	N	Time at risk (years)	Annual rate (Transitions (N)per year at risk)	Approx. 95% Confidence Interval
**Rates of acquiring a bite wound**					
Negative	(N to Bit C) or (N to Bit C) or(Bit P to Bit C) or (N to Bit P)	89	640	0.139	0.113, 0.171
TB positive by culture	(TB-Cul to TB-Cul/Bit C) or (TB-Cul/Bit Pto TB-Cul/Bit C) or (TB-Cul to TB-Cul/Bit P)or (TB-Cul/Bit C to TB-Cul/Bit C)	35	120	0.291	0.209, 0.405
Positive by ELISA	(TB-El to TB-El/Bit C) or (TB-El/Bit Pto TB-El/Bit C) or (TB-El to TB-El/Bit P)or (TB-El/Bit C to TB-El/Bit C)	15	158	0.095	0.057, 0.157
**Rates of becoming positive for ** ***M.bovis* (as detected by culture)**					
No earlier bite woundrecorded	N to (TB-Cul or TB-Cul/Bit C)	14	352	0.040	0.024, 0.067
Earlier bite woundrecorded	(Bit C or Bit P) to (TB-Cul/Bit C or TB-Cul/Bit P)	28	288	0.097	0.067, 0.141
**Rates of being detected as exposed by ELISA result**					
No earlier bite woundrecorded	N to (TB-El or TB-El/Bit C)	17	352	0.048	0.030, 0.078
Earlier bite woundrecorded	(Bit C or Bit P) to (TB-El/Bit C or TB-El/Bit P)	14	288	0.049	0.029, 0.082

The rate of becoming Brock ELISA test positive was not significantly different in badgers that had been bitten from those with no previous evidence of a bite wound: rate ratio = 1.01 (95% CI: 0.50, 2.05, p = 0.98) (Table 3, Figure 1). There was also no evidence that ELISA positive badgers were any more or less likely to be bitten than test negative animals: rate ratio = 0.68 (95% CI: 0.39, 1.18, p = 0.17) (Table 3, Figure 1).

### Trends for All Analyses were Similar when Stratified by Sex

Sensitivity analyses examining the impact of multiple observations per badger indicated that our conclusions were robust to this potential issue. When reducing the dataset to only badgers with one transition (i.e. one contribution to the analysis), rate ratios were similar to those found in the main analysis, although confidence intervals were wide due to low numbers. Dividing our dataset into two equal groups as described above also showed similar results from each group. Of the 14 standard errors of the rates of interest (7 in each group), the majority were approximately √2 times those in the main analysis. Two were slightly higher (2.2 and 2.4 times those in the main analysis). We concluded that there may be some weak evidence that multiple observations would under-estimate the standard errors although given the small p-values of the significant results found, we determined that it was unlikely that this would affect our overall conclusions.

Sensitivity analyses stratifying analyses by age showed similar trends in both age groups.

When excluding culture results obtained from bite wounds from the analyses, similar results were found. The rate of bite wound acquisition in infected badgers (0.268 per year) was 2.45 (95% CI: 1.37, 4.39) times that in uninfected badgers (0.142 per year). The rate of positive *M.bovis* test result in bitten badgers (0.097 per year) was 2.43 (95% CI: 1.28, 4.61) times that in unbitten badgers (0.040 per year).

## Discussion

Our study demonstrates that the association between bite wounding and the progression of TB infection in badgers can potentially be explained by both sequences of events: advanced disease is significantly more likely to occur in bitten badgers whilst animals with advanced disease are significantly more likely to be bitten.

Previous studies have suggested that transmission via biting is possible [10], [20], [21] and although our study cannot confirm this, the results are consistent with transmission during aggressive encounters (as indicated by a bite wound). But the transmission mode could be either inoculation of infectious sputum at the bite site or inhalation of aerosolized bacteria during a close aggressive interaction. The opposite direction of effect is also possible, such that badgers that inflict bites are more likely to become infected if for example biting were to puncture a TB infected lymph node containing large numbers of bacteria [22]. However, this cannot be assessed using our data.

Our findings also suggest that the association between bite wounds and TB infection may be explained by the hypothesis that badgers with advanced disease are more likely to be bitten. *M.bovis* infection in badgers has been associated with poor body condition in animals with advanced disease [14], [23]. Deterioration in body condition would be expected to impact adversely on social status and competitive ability, increasing susceptibility to aggression from other badgers in their resident and neighbouring social groups. Additionally, previous work has shown that culture positive badgers are more likely to range further afield [24], increasing contact with members of other social groups. Our findings provide further evidence for epidemiologically significant behavioural correlates of advanced TB in badgers.

In our analyses involving badgers that were only ELISA test positive, no significant differences were observed. The interpretation of Brock ELISA results is not straightforward because the presence of antibodies to *M.bovis* may be consistent with several potential disease stages [18]. In contrast *M.bovis* isolation by culture indicates the shedding of bacteria and hence active disease, and although it has low sensitivity to detect infection it may provide confirmation of advanced disease [17], [18]. If we assume that ELISA test positive but culture negative status corresponds with an earlier stage of infection, our results provide some evidence that enhanced risks of being bitten occur only once individuals reach a more advanced disease stage. Our results are also consistent with the hypothesis that transmission via biting leads to more rapidly progressive disease as the occurrence of a fresh bite was associated with enhanced likelihood of culture positivity but not a positive ELISA test result. An implication for disease control and an important finding here is that badgers with relatively advanced disease may exhibit distinctive behaviour, as indicated by their propensity to be bitten. As with many diseases a relatively small proportion of the population may make a disproportionately large contribution to disease transmission, as a result of their behaviour [25]. However, it is unclear whether disease management strategies would be more effective if targeted at those individuals being bitten or those doing the biting, as we know little about the latter, or of the consequences of removing either.

There is evidence that some badgers are more or less likely to be trapped [26] which could have introduced bias into our study. However, there was no evidence that TB status or movement (which could influence the frequency of aggressive encounters) affected trappability [26]. While our method of analysis was simple and easy to implement, there are limitations. It was not possible to account for confounding as one might do by inclusion of additional explanatory variables. To reduce the potential effect of age-related variation, we focused on adult badgers (although results for only cubs and yearlings did show similar but non-significant results and results stratifying by age amongst adults showed similar trends). Similar trends were also found stratifying by gender. Variation in the number of captures could have potentially given badgers which had been caught more frequently increased weighting in the analysis and potentially under-estimated standard errors due to the lack of independence by multiple observations from one badger. However, these potential biases were not supported by sensitivity analyses. Additionally, our method makes the approximation that the change from one state to the next occurred at the mid-point between captures; we know of no evidence to support systematic departure from this. The recording of a bite wound and/or infection at each capture is highly dependent on the probabilities of detecting these events. The probability of finding a fresh bite wound or scar is considered to be nearly 100% although a bite wound could have occurred between captures and healed completely. However, culture test sensitivity is considered to be substantially lower than 100% [27]. Therefore, considering the time series of a single badger, if bite wounds and advanced TB infection occurred randomly and independently of one another, we would expect bite wounds to be found earlier in time than advanced TB infection simply on the basis of the probabilities of detection given that there is something to detect. This may help explain our finding of evidence for advanced TB infection following a bite wound but it is unlikely to fully account for it and as described, there is considerable evidence to support transmission of infection during an aggressive encounter.

A further limitation is our inability to identify a causal link. While our results are consistent with both proposed directions of association, they are also consistent with a scenario in which the two events are independently associated with an additional factor such as, for example, increased roaming behavior (affording opportunities for both aggressive interactions and encounters with further sources of infection). However, our study does demonstrate that one direction of effect is not dominant over the other.

The present study provides evidence consistent with the transmission of *M.bovis* infection during aggressive encounters between badgers, and also indicates that infected badgers may exhibit distinctive behaviour themselves and be treated differently by other badgers. The transmission dynamics of TB in badgers are complex and intrinsically linked to their behaviour [28], as supported by this study and previous work (e.g. wider roaming, due to some culling approaches [29], which can increase both the risk of infection [5] and the risk of bite wounding [30]). Bovine TB control remains an important concern for the UK government and the relationships between animal behaviour and infection must be fully considered when designing bovine TB control strategies.
